# Designing Future Clinical Trials for Sepsis-associated Disseminated Intravascular Coagulation

**DOI:** 10.14789/jmj.JMJ24-0010-P

**Published:** 2024-04-18

**Authors:** CHERYL L. MAIER, TOSHIAKI IBA

**Affiliations:** 1 Department of Pathology and Laboratory Medicine, Emory University School of Medicine, Atlanta, GA, USA; 1 Department of Pathology and Laboratory Medicine, Emory University School of Medicine, Atlanta, GA, USA; 2 Department of Emergency and Disaster Medicine, Juntendo University Graduate School of Medicine, Tokyo, Japan; 2 Department of Emergency and Disaster Medicine, Juntendo University Graduate School of Medicine, Tokyo, Japan

**Keywords:** sepsis, disseminated intravascular coagulation, clinical trial, anticoagulants, composite endpoint

## Abstract

Defining success in a clinical trial is not necessarily a straightforward task, especially when the target population is critically ill patients where few agents have demonstrated effectiveness. This has been the case for trials of anticoagulation in patients with sepsis-associated disseminated intravascular coagulation (DIC), which have generally examined patients with severe sepsis but not specifically DIC. Limitations of existing studies include inadequate anticoagulant doses and delayed initiation of treatment. Furthermore, 28-day mortality has been adopted as the primary endpoint but is affected by a panoply of factors other than anticoagulant therapies and may not be the most relevant measure. Future trials must address several current limitations in order to improve our understanding of the role of anticoagulation in patients with sepsis-associated DIC.

## Patient screening

Patient screening is an essential component of clinical trial design yet remains a major challenge for clinical trials of sepsis-associated DIC. This may, in large part, reflect the fact that a priori screening for disseminated intravascular coagulation (DIC) in sepsis is not routinely performed outside of Japan. In addition, most diagnostic criteria for DIC consist of multiple laboratory parameters, including platelet count, prothrombin time, fibrin/fibrinogen degradation products (i.e., D-dimers), and fibrinogen, which may not be routinely followed in critically ill patients. The sepsis-induced coagulopathy (SIC) criteria, composed of only platelet count and prothrombin time-international normalized time (PT-INR) as laboratory parameters alongside the sequential organ failure assessment (SOFA) score^1)^, offer a more feasible method for patient identification. Notably, the use of the SIC criteria has been shown to capture almost all cases that progress to overt DIC, and its scoring is suitable for screening^2)^. Therefore, we recommend patient screening for clinical trial inclusion using the SIC criteria to ensure appropriate candidates are not overlooked.

## Patient selection

Defining the proper patient population is critical for success in clinical trials. Most studies examining the effects of anticoagulant therapies have been conducted in patients with severe sepsis; however, none of them have shown benefit in this population. Nevertheless, anticoagulant therapy has been found to be effective in patients with sepsis-associated DIC^3)^, and the effect of anticoagulant therapy has been more prominent in patients with greater disease severity^4)^. These reports examined studies that adopted 28-day mortality as a primary endpoint. When all-cause mortality is the endpoint, it is reasonable to think that the study protocol should specify a target mortality. We recently proposed a stepwise classification strategy using a decision tree with variables including baseline SOFA score, antithrombin activity, underlying disease, sex, and age to identify patients with an estimated mortality rate ranging from 20 to 40%^5)^. This type of approach is recommended in the design of future clinical trials.

## Anticoagulants

Antithrombin concentrate and recombinant thrombomodulin are frequently used in Japan. Nevertheless, the effectiveness of either agent has not been proven in a randomized controlled trial (RCT). Antithrombin is the most abundant and arguably the most important physiological anticoagulant, inhibiting several key enzymes of the coagulation system. Antithrombin activity is significantly decreased in sepsis-associated DIC, both from physiologic consumption and permeability-related extravasation; thus, supplementation is considered to be a rational approach. Surprisingly, and despite suggested benefit in smaller studies, the largest RCT of antithrombin in sepsis-associated DIC failed to demonstrate efficacy^6, 7)^. Multiple causes were suspected regarding the failure of the RCT, highlighting the need for additional consideration in the development of future trials.

Similar findings have been reported for recombinant thrombomodulin in sepsis-associated DIC. Thrombomodulin is a transmembrane anticoagulant expressed on the surface of the vascular endothelium. Thrombomodulin activates protein C by binding with thrombin, and activated protein C inhibits coagulation factors Va and VIIIa. Although a positive effect of recombinant thrombomodulin was reported in patients with DIC^8)^, the SCARLET trial, the first RCT that targeted sepsis-associated coagulopathy, failed to demonstrate efficacy^9)^.

## Dose and timing

The dose and timing of anticoagulant administration are anticipated to be critical in mitigating the disease progression of sepsis-associated DIC. Unfortunately, the optimal dose may not yet be determined, as is the case for antithrombin concentrate. We previously examined the relationship between antithrombin dose and patient survival, finding that 3,000 IU/day of antithrombin administration for three days was associated with superior survival compared to 1,500 IU/day for three days^10)^. In addition, Akahoshi et al.^11)^ have examined any relationship between antithrombin activity post-supplementation and patient outcomes, reporting significantly higher survival rates in patients who achieved antithrombin activity ≥ 80%. Since the post-treatment antithrombin activity did not reach this level in many of the patients in the aforementioned RCT of antithrombin supplementation, we hypothesize that a standard dosage in Japan (1,500 IU/day, and lower than the other countries) might have been insufficient to see a benefit, and a higher dose was likely required.

Inadequate timing in the initiation of anticoagulant therapy is another important issue to consider. In the SCARLET trial, 28-day mortality improved by only 2.6% in the intention-to-treat population allocated to recombinant thrombomodulin. However, more than 20% of the patients recovered before treatment initiation, and subgroup analysis in patients who fulfilled entry criteria at baseline revealed a reduction in 28-day all-cause mortality by 5.4%^9)^. While it is understandable that obtaining informed consent takes time, especially when patients are critically ill, it is necessary to interpret RCT results in light of relevant limitations.

## Endpoint setting

Traditionally the gold standard for endpoint analysis in sepsis trials has been 28-day mortality. Since mortality is influenced by numerous factors beyond the target therapies, a recent sepsis trial investigating the impact of Vitamin C instead used the change in organ failure assessed by SOFA score as the primary outcome. Interestingly, despite no significant differences in that primary endpoint, patients infused with Vitamin C did demonstrate a significant reduction in 28-day all-cause mortality^12)^. The use of a composite endpoint as a primary outcome has been applied in recent clinical trials^13)^. In this method various events observed in the disease are amalgamated as outcomes. For example, in the case of DIC, in addition to death, improvements in organ function and DIC resolution can be considered for the single study outcome. The composite endpoint offers several advantages, such as improving the ability to detect differences, allowing smaller sample sizes, and shortening study completion times^14)^. Moreover, its usefulness has already been demonstrated in trials targeting COVID-19- associated coagulopathy^15)^.

## Conclusion

**Figure 1:**
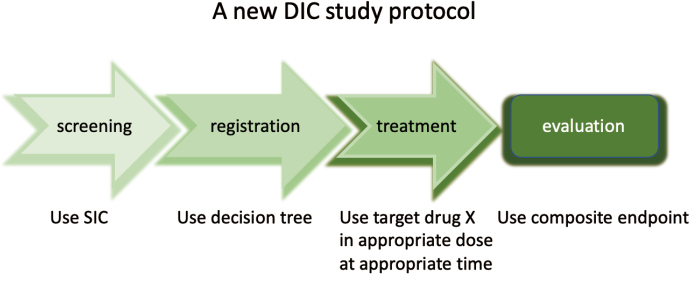
Proposal of a new study protocol examining the effects of anticoagulants for sepsis-associated DIC It is important to look back at the factors that caused the failure of clinical trials and refine our protocols going forward. Major contributing factors have included the lack of patient screening, absence of mortality estimation, inappropriate use of the target agents, and inadequate evaluation. DIC: disseminated intravascular coagulation, SIC: sepsis-induced coagulopathy

There are multiple reasons for the failure of clinical trials to define therapeutic efficacy despite mechanistic rationale for why a drug may be beneficial. Was the patient’s eligibility screened appropriately? Were the inclusion criteria properly set? Was the target agent properly used? Was the endpoint adequate? Given the nuance related to each of these questions and the fact that any potential benefit from a single agent is likely not as measurable as hoped, it is important to define and refine our clinical trial designs continuously (Figure 1).

## Funding

No funding was received.

## Author contributions

CLM and TI wrote and reviewed the manuscript. Both authors read and approved the final manuscript.

## Conflicts of interest statement

The authors declare that they have no conflict of interest.

## References

[1] Iba T, Nisio MD, Levy JH, Kitamura N, Thachil J: New criteria for sepsis-induced coagulopathy (SIC) following the revised sepsis definition: a retrospective analysis of a nationwide survey. BMJ Open, 2017; 7: e017046. 10.1136/bmjopen-2017-017046 PMC5623518 28963294

[2] Thachil J, Iba T: Designing the diagnostic criteria for disseminated intravascular coagulation (DIC). Juntendo Medical Journal, 2023; 69: 1-3. 10.14789/jmj.JMJ23-0038-P PMC11153069 38855069

[3] Umemura Y, Yamakawa K: Optimal patient selection for anticoagulant therapy in sepsis: an evidence-based proposal from Japan. J Thromb Haemost, 2018; 16: 462-464. 29316171 10.1111/jth.13946

[4] Yamakawa K, Gando S, Ogura H, et al: Identifying Sepsis Populations Benefitting from Anticoagulant Therapy: A Prospective Cohort Study Incorporating a Restricted Cubic Spline Regression Model. Thromb Haemost, 2019; 119: 1740-1751. 31408900 10.1055/s-0039-1693740

[5] Iba T, Maier SL, Tanigawa T, Levy JH: Risk stratification utilizing Sequential Organ Failure Assessment (SOFA) score, antithrombin activity, and demographic data in sepsis-associated disseminated intravascular coagulation (DIC). Sci Rep, 2023; 13: 22502. 38110515 10.1038/s41598-023-49855-y PMC10728127

[6] Tagami T, Matsui H, Horiguchi H, Fushimi K, Yasunaga H: Antithrombin and mortality in severe pneumonia patients with sepsis-associated disseminated intravascular coagulation: an observational nationwide study. J Thromb Haemost, 2014; 12: 1470-1479. 24943516 10.1111/jth.12643

[7] Warren BL, Eid A, Singer P, et al: Caring for the critically ill patient. High-dose antithrombin III in severe sepsis: a randomized controlled trial. JAMA, 2001; 286: 1869-1878. 11597289 10.1001/jama.286.15.1869

[8] Saito H, Maruyama I, Shimazaki S, et al: Efficacy and safety of recombinant human soluble thrombomodulin (ART-123) in disseminated intravascular coagulation: results of a phase III, randomized, double-blind clinical trial. J Thromb Haemost, 2007; 5: 31-41. 17059423 10.1111/j.1538-7836.2006.02267.x

[9] Vincent JL, Francois B, Zabolotskikh I, et al: Effect of a Recombinant Human Soluble Thrombomodulin on Mortality in Patients With Sepsis-Associated Coagulopathy: The SCARLET Randomized Clinical Trial. JAMA, 2019; 321: 1993-2002. 31104069 10.1001/jama.2019.5358 PMC6547077

[10] Iba T, Saitoh D, Wada H, Asakura H. Efficacy and bleeding risk of antithrombin supplementation in septic disseminated intravascular coagulation: a secondary survey. Crit Care, 2014; 18: 497. 25220851 10.1186/s13054-014-0497-x PMC4182824

[11] Akahoshi T, Kaku N, Shono Y, et al: Antithrombin Activity Levels Following Recombinant Antithrombin Gamma Therapy in Patients with Sepsis-Induced Disseminated Intravascular Coagulation. Clin Appl Thromb Hemost, 2022; 28: 10760296221135790. 36380520 10.1177/10760296221135790 PMC9676283

[12] Fowler AA 3rd, Truwit JD, Hite RD, et al: Effect of Vitamin C Infusion on Organ Failure and Biomarkers of Inflammation and Vascular Injury in Patients With Sepsis and Severe Acute Respiratory Failure: The CITRIS-ALI Randomized Clinical Trial. JAMA, 2019; 322: 1261-1270. 31573637 10.1001/jama.2019.11825 PMC6777268

[13] Bakal JA, Westerhout CM, Armstrong PW: Impact of weighted composite compared to traditional composite endpoints for the design of randomized controlled trials. Stat Methods Med Res, 2015; 24: 980-988. 22275378 10.1177/0962280211436004

[14] McCoy CE: Understanding the Use of Composite Endpoints in Clinical Trials. West J Emerg Med, 2018; 19: 631-634. 30013696 10.5811/westjem.2018.4.38383 PMC6040910

[15] Cools F, Virdone S, Sawhney J, et al: Thromboprophylactic low-molecular-weight heparin versus standard of care in unvaccinated, at-risk outpatients with COVID-19 (ETHIC): an open-label, multicentre, randomised, controlled, phase 3b trial. Lancet Haematol, 2022; 9: e594-e604. 35779560 10.1016/S2352-3026(22)00173-9 PMC9243570

